# Remarks on the "Light Treatment" of Lupus, Vulgaris and Erythematosus, and Rodent Ulcer, &c.

**Published:** 1903-03

**Authors:** A. J. Harrison, W. Kenneth Wills

**Affiliations:** Physician to the Skin Department of the Hospital


### REMARKS ON THE "LIGHT TREATMENT" OF LUPUS, VULGARIS AND ERYTHEMATOSUS, AND RODENT ULCER, &c.,

### AS CARRIED OUT AT THE BRISTOL GENERAL HOSPITAL SINCE DEC. 9TH, 1901, TO THE END OF DEC., 1902.

BY

A. J. Harrison, M.B. (Lond.),

*Physician to the Skin Department of the Hospital,*

AND

W. Kenneth Wills, M.A., M.B., B.C. (Cantab.).

---

It will be observed that the period of time covered by the treatment is a little more than twelve months. At first only two lamps were used—Lortet-Genoud, made by Marshall and Woods, of London; but from May 23rd, 1902, two other lamps (constructed by Leslie Miller, of London) were added. The current used has been an interrupted one—five to ten ampères, about twelve volts, and the time of each sitting at the lamps has been from five to twenty minutes. Several of the patients have had two, three, or four sittings on each day of their attendance, and especially where the extent of the affected parts was great, or the disease was situated in several parts of the body, —one part being treated at one sitting, another part at another sitting.

In many instances X-rays have been employed: often in cases of lupus vulgaris, where the parts were ulcerated, and very especially so in cases of lupus erythematosus, rodent ulcer, acne pustulata, and Paget's disease.

During the time there have been treated 42 cases of lupus vulgaris, 3 of lupus erythematosus, 12 of rodent ulcer, 1 of the late stage of actino-mycosis, 3 of acne pustulata, 1 of adenoma sebaceum, 2 of Paget's disease, and 1 of advanced carcinoma of the right breast and axilla.

Taking the lupus vulgaris cases first—16 males and 26 females have been treated, their ages varying from 4½ years to 73, and the duration of the disease from 1 to 45 years. From the above statements it will be readily conceived that many of the cases have been under various kinds of treatment for years, and many of them had been treated with caustic applications, or scraped, once or many times. Many of them, therefore, have been the accumulation, so to speak, of years. It was observed that the cases responded best to treatment that had never been more or less severely dealt with.

Before giving fuller details of some of the cases, the results so far obtained may be briefly summarised :—

| | |
|---|---|
| Improved and continuing under treatment ... | 26 |
| Relieved—to be kept under observation ... | 5 |
| Relieved—in one or more parts, whereas other parts have to be treated... ... ... ... ... | 4 |
| Not improved ... ... ... ... ... ... ... | 1 |
| Unsatisfactory, through non-attendance ... | 2 |
| Cases under treatment, but time too little to judge of effect ... ... ... ... ... ... | 4 |

Further details of some of the cases will be interesting :—

E. H., male, aged 21. Commenced treatment on December 10th, 1901.

*Condition.*—Very great thickening of upper lip, which is very red and much ulcerated, and hangs protrudingly over the lower lip. The alæ and septum nasi are ulcerated, and there is considerable loss of tissue; the nasal orifices are very contracted. The mucous membrane of the nose is involved, and this gives rise to an irritating discharge, which frets and ulcerates the parts below. There are numerous outlying nodules on the cheeks and nose. Has epiphora.

*Duration.*—Six years. Disease commenced on margin of right nostril. As the "light treatment" has not much effect upon mucous membranes, iodine solutions with cocaine were applied to the inside of the nostrils, which diminished the irritating discharge, and soon produced great improvement. Thyroid extract gr. v., twice daily, was also given for several weeks to reduce thickening.

*Present condition.*—There is no discharge from the nostrils; the upper lip is nearly as small as it should be; the ulceration is nearly healed; the redness is much less; and the nodules have disappeared almost entirely—some of them, quite.

E. B., female, aged 56.

*Condition.*—The whole of the face, from the hair on forehead to the chin, and from ear to ear, is one mass of darkly-stained deposits, with more or less scar-tissue intermingled. The *present* nose is stained a reddish-brown colour, which does not disappear upon pressure. Both lower eyelids are drawn down, exposing the mucous membrane.

*History.*—Forty-five years ago the disease commenced on the left edge and inside of nostril, then gradually spread. The parts were never scraped, but Mr. Cross skin-grafted the lower eyelids. Later on in life a large part of the nose perished, so about thirteen years ago the late Mr. Greig Smith restored the nose by forming one from the right arm. He was nothing if not artistic, and the symmetrical and consistent pug-nose which he designed was as suitable to the face as if Nature, in her best mood, had created it.

At that time Dr. Harrison happened to see this case, with Mr. Greig Smith, in the Infirmary, and little did he then anticipate that this excellent plastic work would be, in the course of time, mysteriously attacked by the devouring element, and become an object for "light treatment" at the Bristol General Hospital. Surely this is one of the most curious cases on record!

*Present condition.*—This case has been well treated; the woman has attended most regularly; and although the results have been slow, they are very hopeful.

J. W., male, aged 32. Commenced "light treatment" on January 9th, 1902.

*Condition.*—The whole of the face and neck, from forehead to below the clavicles, is one mass of erythematous scaling or slightly ulcerated surfaces, showing little tendency to heal, and displaying a general light-brown staining. The left eye has been involved, leading to corneal opacity and almost blindness, and there is some opacity of the right cornea, but the sight is good. The eyelids and lips are much affected. There are two lupus patches on the back, one of them about four inches long, and they are very tender at times.

*History.*—When nine years old he had what was said to be an attack of erysipelas of the face (most probably erythematous eczema), caused by keeping on wet clothes for many hours, when he and other boy companions made a day's nutting of it in the country.

After the attack disappeared a red spot showed itself over the left eyebrow, and from this gradually, but too inevitably, the lupus developed.

He was brought up to the coach-painting trade, and he became a good and careful workman. As time went on the disease began to interfere with his work, and he became such an object that he rarely went out until dusk, and life was rather unhappy and miserable. He was well known to many practitioners who frequented Clifton Wood, where he lived. About twelve years ago he came under Dr. Harrison's care, and by treating him with lotions of hyposulphite of soda and dilute hydrochloric acid for some months he improved so much that he and several other patients, similarly treated, were shown at the annual meeting of the British Medical Association, at Nottingham, in 1892, as good examples of this mode of treatment. So much did he improve that he was able to follow his coach-painting occupation, and could earn from twenty to forty shillings a week at it.

Some years afterwards, as he did not get much better—*i.e.* as above stated,—he came into the Bristol General Hospital to be treated with tuberculin. He reacted in a very remarkable manner. Some improvement was made, but it was not maintained.

Later on he was attacked with Lichen ruber planus, one of the most marked and extensive developments of the complaint ever seen; it got well, but, unfortunately, without taking the lupus with it in its disappearing train.

For the last year or two—partly from some indifference on his part, and from being, consequently, rather lost sight of—he had been going back. The "light treatment" had scarcely descended to the plains of human ailments, being chiefly known (probably) in Alpine heights by its tendency to produce so-called frost-bites and skin-scaled faces. He commenced the "light treatment" on January 29th, 1902, and has been most regular and indefatigable in his attendance, with the satisfactory result that there is generally much clearing up of the face, there is much less scaling, and the skin is almost white in patches.

J. McC., male, aged 18. Admitted into the hospital for "light treatment" May 3rd, 1902.

*Condition.*—The first thing which strikes an observer on looking at the weakly and undersized boy is the peculiar constricted mouth, which is raw and ulcerated all round the orifice of the lips in a ring, and cannot be opened for more than a quarter of an inch. The teeth are very irregular and foul, but not "pegged." Inside the mouth the mucous membrane is ulcerated with lupus deposits; the eyelids are ectropic and bound to the conjunctiva in places, and speckled with numerous small cysts. The aspect looks like lupus attacking a boy afflicted with congenital syphilis.

*History.*—Little is known of his history: he is an orphan in charge of the Bath Guardians, who have obtained his admission into the hospital to give him the benefit of the "light treatment."

*Treatment.*—He has thoroughly undergone the "light treatment," and has had internal remedies as well, with the result that his mouth is almost completely healed, the eyelids are better, and the mouth inside is much improved by the removal of the useless teeth, or some of them, and the future looks more hopeful for him.

F. H., female, aged 30. Admitted August 4th, 1902.

*Condition.*—On both cheeks, nostrils, tip of nose, and upper and lower lips are masses of hypertrophied lupous tissue. The lips, especially the lower, are pendulous and very ulcerated, and almost repulsive in appearance; the tongue, roof of mouth, and gums are also much affected; the left temple and the chin are involved; and about four inches above the left elbow is a lupus patch. The left little finger is affected with tubercular dactylitis, and has discharging sinuses.

*History.*—Began about twelve years ago on the right arm near the elbow. It was treated with ointments, and healed up, leaving only a purplish-looking mark. A few years ago she was under Dr. Harrison's care, when the face was affected, but not so severely as now, and the left elbow. Two years ago she lost use in the right arm and leg, which came on with a shaking sensation, but there was no paresis. After this attack the face and mouth became much worse. The present appearance of the lips and mouth is really very repulsive.

*General condition.*—Spiritless and rather depressed, but takes food well. General health, otherwise, fairly good, and there has been no loss of weight.

*Treatment.*—As she lived away from Bristol she was admitted as an in-patient. "Light treatment" for sixty-four days—two or more places at a time, fifteen minutes each sitting. Lotions applied at first, and X-rays on three occasions greatly improved the ulcerated condition of the lips. She also took with benefit thyroid extract. As she wished to go home for a time, she left the hospital on October 17th, 1902, very much improved; but unquestionably the treatment should be continued.

Three cases of lupus erythematosus have been under observation. This seems a very small proportion of cases; but this complaint in its usual sense is by no means common in this part of the country. Two of the cases improved, but one of these not much until the X-rays were also used. The third case was unsatisfactory, through indifferent attendance.

Twelve cases of rodent ulcer have been treated, chiefly with X-rays, and the results in most of these cases have been markedly beneficial. Seven occurred in males and twenty-five in females. The youngest age is 23; but there was a little doubt about this case, and being a seafaring man he disappeared. The next youngest was a female, 36, who had an ulcer just outside the outer canthus of the left eye, and there was then a history of nine years' duration. The greatest age was in a female, 73, with six months' history, and a man, 72, with fifteen years' history, which case is given more fully just below. Some of the cases improved very rapidly.

The case of H. L., a farmer, aged 72, is one of the most remarkable we have ever seen.

*Condition.*—Extensive destruction of superior maxilla, nose, and cheeks. The upper lip is entirely destroyed, and the superior surface of the maxilla, the central incisor teeth being exposed for their whole length. Half of the nose (septum and alæ) is gone. The ulceration is extending to the inner canthus of left eye, downwards to lower lip on the left side, and into both cheeks—the left more involved than the right.

*History.*—Fifteen years' duration. Began as a small spot between cheek and nose on left side.

*General health.*—In spite of this awful affliction, he has most wonderful pluck, and says he "feels as young as 40."

*Treatment.*—He has been treated with X-rays since October 25th—eighteen times to present date—with the result that after the first few exposures he had considerable pain: later, this disappeared, and now he gets occasional attacks of numbness in the face and left eye. The extensive, ulcerated, and weeping surface is now quite dry and no longer foul, and epithelium can be detected growing in from the edges so as to cover over some of the raw parts.

Of these twelve cases of rodent ulcer, five are now under observation before being pronounced cured. One of these latter is of interest.

E. C., aged 73, female, on February 22nd, 1902, presented a typical rodent ulcer, of the size of half-a-crown, on the right side of the upper lip, which was puckered up so as permanently to show the right upper canine tooth. The ulcer extended upwards on to the cheek and encroached upon the ala of the nose. The whole thickness of the lip was involved, but there was no perforation. The edges were dense, everted, and in places pigmented a dark, bluish-black, bringing to mind a melanotic tumour. There was very little discharge. It was of twenty years' duration and had had no surgical treatment. Since February, 1902, patient has had thirty-eight applications of the rays—frequently at long intervals, owing to her being frail and often unable to attend the hospital. At present the ulcer has completely healed over, and the raised edges, which gave more trouble to remove than the ulcer itself, have now disappeared; though on her last appearance there was a little scaling where the last part of the edges had been. There is some puckering of the scar left, but it certainly is no more marked than it was when first seen.

Some other cases of interest are three instances of acne vulgaris, which have not yielded to ordinary treatment. Considering the use of the X-rays in ulcerated lupus, and indeed in all cases of ulceration, it was thought that a good effect might follow in these cases; and although it is currently reported that the X-rays have no bactericidal properties, yet undoubtedly their use has been justified. Together with this treatment, the comedones were expressed energetically and pustules punctured; but in the first of these cases there was abundant evidence that the good effect was not entirely due to this adjuvant treatment.

W. T., aged 18, male, had extensive pustular acne upon his forehead, nose, and cheeks, which, for the last four years, had rendered him such a spectacle that he had had the greatest difficulty to obtain any employment at all. He was treated on twenty-two occasions between August 13th and December 12th, about twice a week, and at present he shows no pustules or comedones, and his skin, though much scarred from old pustules, is supple and much smoother.

E. H., male, aged 25, was admitted on October 13th for very extensive and severe acne pustulata of the back, from the back to the sacrum. He had suffered from it for nearly nine years, and in spite of active expression of comedones and pustules and antiseptic treatment, was none the better. He could carry nothing on his back, and sometimes could hardly bear his clothes on. Many of the pustules had undermined the skin in all directions, often joining one another. In a fortnight, after four applications of the rays, he was able for the first time to bear hard pressure on the back, and could wear his clothes in comfort. More pustules appeared later; but gradually the skin is growing more healthy, and the large craters left by the undermining pustules are nearly all healed up, after thirteen applications extending over three and a-half months. An occasional pustule is seen now, but is instantly dealt with.

The third case, C. M., male, aged 17, is at present incomplete; but enough has been done to show that this case will react as the others have done. Already patches of skin tracked with small sinuses and crowded with comedones are clearing and healing.

The cases of Paget's disease are very interesting and have been greatly benefited, but the fuller history must be reserved for another summary.

